# Critical switching current density induced by spin Hall effect in magnetic structures with first- and second-order perpendicular magnetic anisotropy

**DOI:** 10.1038/s41598-017-15681-2

**Published:** 2017-11-10

**Authors:** Seok Jin Yun, Kyung-Jin Lee, Sang Ho Lim

**Affiliations:** Department of Materials Science and Engineering, Korea University, Seoul, 02841 South Korea

## Abstract

In this study, we derive analytical expressions for the critical switching current density induced by spin Hall effect in magnetic structures with the first- and second-order perpendicular magnetic anisotropy. We confirm the validity of the expressions by comparing the analytical results with those obtained from a macrospin simulation. Moreover, we find that for a particular thermal stability parameter, the switching current density can be minimized for a slightly positive second-order perpendicular magnetic anisotropy and the minimum switching current density can further be tuned using an external magnetic field. The analytical expressions are of considerable value in designing high-density magnetic random access memory and cryogenic memory.

## Introduction

The strength of the perpendicular magnetic anisotropy (PMA) is an important parameter that affects the performance of spin devices, such as magnetic random access memory (MRAM)^1^, magnetic sensors^2,3^, and spin torque oscillators^4–7^. Magnetic tunnel junctions (MTJs) with PMA are promising as storage cells in high-density MRAM because of their high thermal stability and low switching current density^1,8^. Recently, spin devices using magnetic structures with both first- and second-order PMA have attracted considerable interest owing to their novel properties^9–16^. For MRAM where the magnetization is switched by the spin-transfer torque (STT), the magnetization switching can be fast, without heat assistance and incubation time, by using MTJs with an easy-cone state, which can be formed when the second-order PMA is considerably strong^9,10^. Recently, it was demonstrated that in NM/FM bilayers (NM and FM denote the nonmagnetic and ferromagnetic, respectively), an in-plane current flowing through an NM layer can induce a spin torque acting on the adjacent FM layer, which is sufficient to reverse its magnetization^17–23^. This spin torque is completely different from the STT generated by an out-of-plane current flowing through the MTJ stack. The spin torque by the in-plane current is known as a spin–orbit torque (SOT) generated by the spin Hall effect (SHE)^18,19,24–26^. The SOT in NM/FM bilayers attracted intense interest because the SOT-MRAM can be operated at an ultrafast speed and therefore, is a promising candidate for replacing conventional static random access memory (SRAM)^27–29^. A cryogenic memory cell integrated with a Josephson junction circuit can be another promising application of NM/FM bilayers utilizing the ultrafast SOT switching^28,30–32^. Although numerous studies have been conducted for examining the effects of various parameters such as applied current pulse width and external magnetic field on the performance of the SOT switching, no investigations have been performed on the effects of the second-order PMA, which is particularly important in cryogenic applications where the second-order PMA is strong and sometimes dominant over the first-order PMA^27,28,33^. In this study, analytical expressions for the SHE-induced critical switching current density (*J*_c_) are derived in magnetic structures with the first- and second-order PMA, and their validity is tested by comparing the analytical results with those obtained from a macrospin simulation. Then, the analytical expressions are used for systematically examining the SHE-induced *J*_c_ as functions of first- and second-order PMA strengths. Finally, the analytical expressions are utilized to optimize the SHE-induced *J*_c_ and the thermal stability parameter, which are important device parameters related to the power consumption and data duration time, respectively.

## Results

### Easy-cone state and its magnetization switching behavior

**Figure 1:**
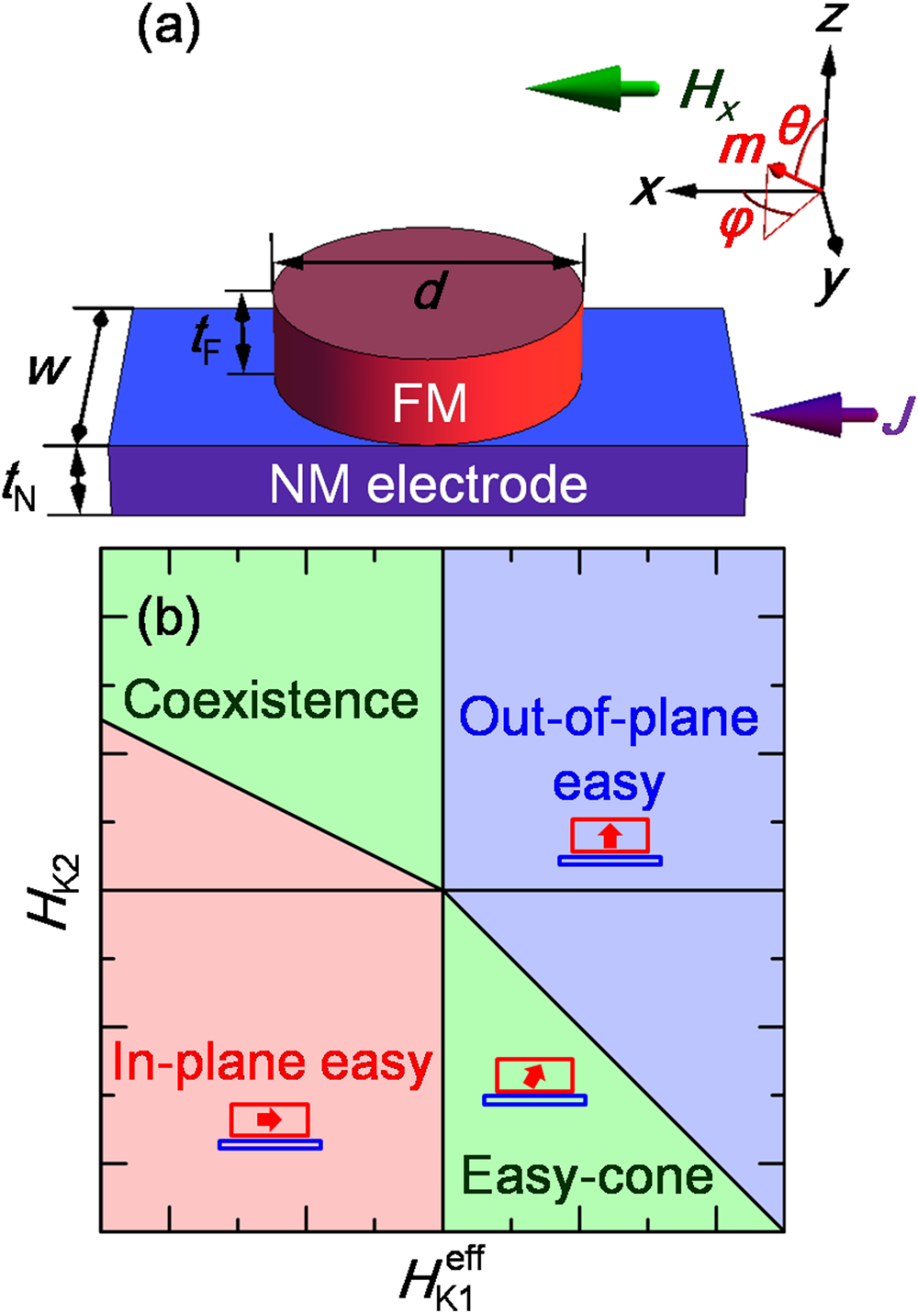
(**a**) Schematic showing NM/FM bilayered structure together with definition of *x*-, *y*-, and *z*-axes and magnetization angles of *θ* and *φ*. The directions of *H*_*x*_ and *J* are also indicated. (**b**) Phase diagram showing stable magnetic states as functions of *H*_K1_^eff^ and *H*_K2_.

A schematic of the SHE-based device examined in this study is shown in Fig. 1(a), which comprises an NM electrode and an FM nanodot. Moreover, the geometric parameters such as the width (*w*) and the thickness (*t*_N_) of the NM electrode and the diameter (*d*) and the thickness (*t*_F_) of the FM nanodot, together with the definition of Cartesian (*x*-, *y*-, and *z*-) axes and the polar (*θ*) and azimuthal (*φ*) angles of the normalized magnetization vector (***m***), are shown in the figure. The geometrical parameters used were *d* = 30 nm, *w* = 30 nm, *t*_F_ = 1 nm, and *t*_N_ = 2 nm. The NM electrode serves as a switching current path, and the FM nanodot serves as a magnetic free layer. A tunneling barrier, such as MgO or AlO_*x*_, and a pinned FM layer can be added on top of the FM nanodot for producing an SOT-MTJ cell. In the case of a cryogenic memory cell, a Josephson junction serving as a stray magnetic field sensor can be added on top of the FM nanodot. The magnetic easy axis of the FM nanodot was formed along the *z*-axis or the direction slightly canted from the axis due to the second-order PMA. An external magnetic field along the *x*-axis (*H*_*x*_) was applied to make the SOT switching deterministic^17,19,27^. A phase diagram showing the magnetic easy axis of the FM is shown in Fig. 1(b) as a function of the effective first-order PMA field (*H*_K1_^eff^) and second-order PMA field (*H*_K2_). Here, *H*_K1_^eff^ and *H*_K2_ are defined from the equation for the PMA energy (*E*_PMA_)^34–36^:

1 $${E}_{{\rm{PMA}}}=-{K}_{1}^{{\rm{eff}}}{V}_{{\rm{F}}}{\cos }^{2}\theta -{K}_{2}{V}_{{\rm{F}}}{\cos }^{4}\theta .$$

Here, *K*_1_^eff^ is the effective first-order PMA energy density that considers the demagnetizing term: *K*_1_^eff^ = *K*_1_ − *N*_d_ *M*_S_^2^/2 (*K*_1_, *N*_d_, and *M*_S_ are the first-order PMA energy density, demagnetizing factor, and saturation magnetization, respectively). *K*_2_ is the second-order PMA energy density, and *V*_F_ is the volume of the FM. The following equations were used for obtaining *H*_K1_^eff^ and *H*_K2_: *H*_K1_^eff^ ≡ 2*K*_1_^eff^/*M*_S_; *H*_K2_ ≡ 4*K*_2_/*M*_S_. Using equation (1), it is a straightforward task to construct the phase diagram shown in Fig. 1(b). The magnetic easy axis is along the *z*-axis (out-of-plane easy state) when *H*_K1_^eff^ > 0 and *H*_K2_/*H*_K1_^eff^ ≥ −1, and it is canted slightly from the *z*-axis (easy-cone state) when *H*_K1_^eff^ > 0 and *H*_K2_/*H*_K1_^eff^ < −1. The equilibrium polar angle of the magnetization (*θ*_E_) is 0 and *π* for the out-of-plane easy state; whereas, for the easy-cone state, *θ*_E_ can be determined from the relation cos^2^ *θ*_E_ = −*H*_K1_^eff^/*H*_K2_^33,37,38^.

For the macrospin simulation, the modified Landau−Liftshitz−Gilbert equation including a damping-like SOT was numerically solved^19,27^.

2 $$\frac{\partial {\boldsymbol{m}}}{\partial t}=-\gamma {\boldsymbol{m}}\times {{\boldsymbol{H}}}_{{\rm{eff}}}+\alpha {\boldsymbol{m}}\times \frac{\partial {\boldsymbol{m}}}{\partial t}+\gamma {c}_{J}{\boldsymbol{m}}\times ({\boldsymbol{m}}\times \hat{y}),$$

3 $${{\boldsymbol{H}}}_{{\rm{eff}}}={H}_{{\rm{K1}}}^{{\rm{eff}}}\,\cos \,\theta \,\hat{z}+{H}_{{\rm{K2}}}{\cos }^{3}\theta \,\hat{z}+{H}_{x}\hat{x}.$$

Here, the symbols *γ*, *α*, and *c*_*J*_ denote the gyromagnetic ratio, the damping constant, and the strength of the damping-like SOT, which is shown by the relation *c*_*J*_ = (*ħ*/2*e*)(*θ*_SH_ *J*/*M*_S_ *t*_F_) (*ħ* is the Planck constant, *e* is the electron charge, *θ*_SH_ is the spin Hall angle, *J* is the in-plane current density, and *M*_S_ is the saturation magnetization)^39^. The temporal variation of the normalized magnetization vector (∂***m***/∂*t*) is described as the summation of three terms: the precessional torque induced by the effective field (***H***_eff_), the damping torque, and the damping-like SOT. This work focuses on the damping-like SOT predominantly; however, some results related to the field-like SOT, which is considerably large in some cases^20–22^, are described in Supplementary Section 4. The direction of the SOT is perpendicular to that of *H*_K1_^eff^ and *H*_K2_, whereas the direction of the STT is collinear to that of *H*_K1_^eff^ and *H*_K2_. Because of this, there are two main differences in the switching behavior. First, the SOT indirectly competes with the damping torque originating from *H*_K1_^eff^ and *H*_K2_, whereas the STT competes directly with the damping torque. Owing to its indirect competition, the SOT switching is significantly faster than STT one. This feature of indirect competition also makes the critical switching current density to be independent of *α*. Second, the SOT manipulates ***m*** to be along + *y* (or −*y*) and therefore, the SOT switching is stochastic. To acquire a deterministic SOT switching, it is necessary to apply the field component *H*_*x*_; the precessional torque due to *H*_*x*_ causes ***m*** to be tilted slightly to + *z* (or −*z*).

**Figure 2:**
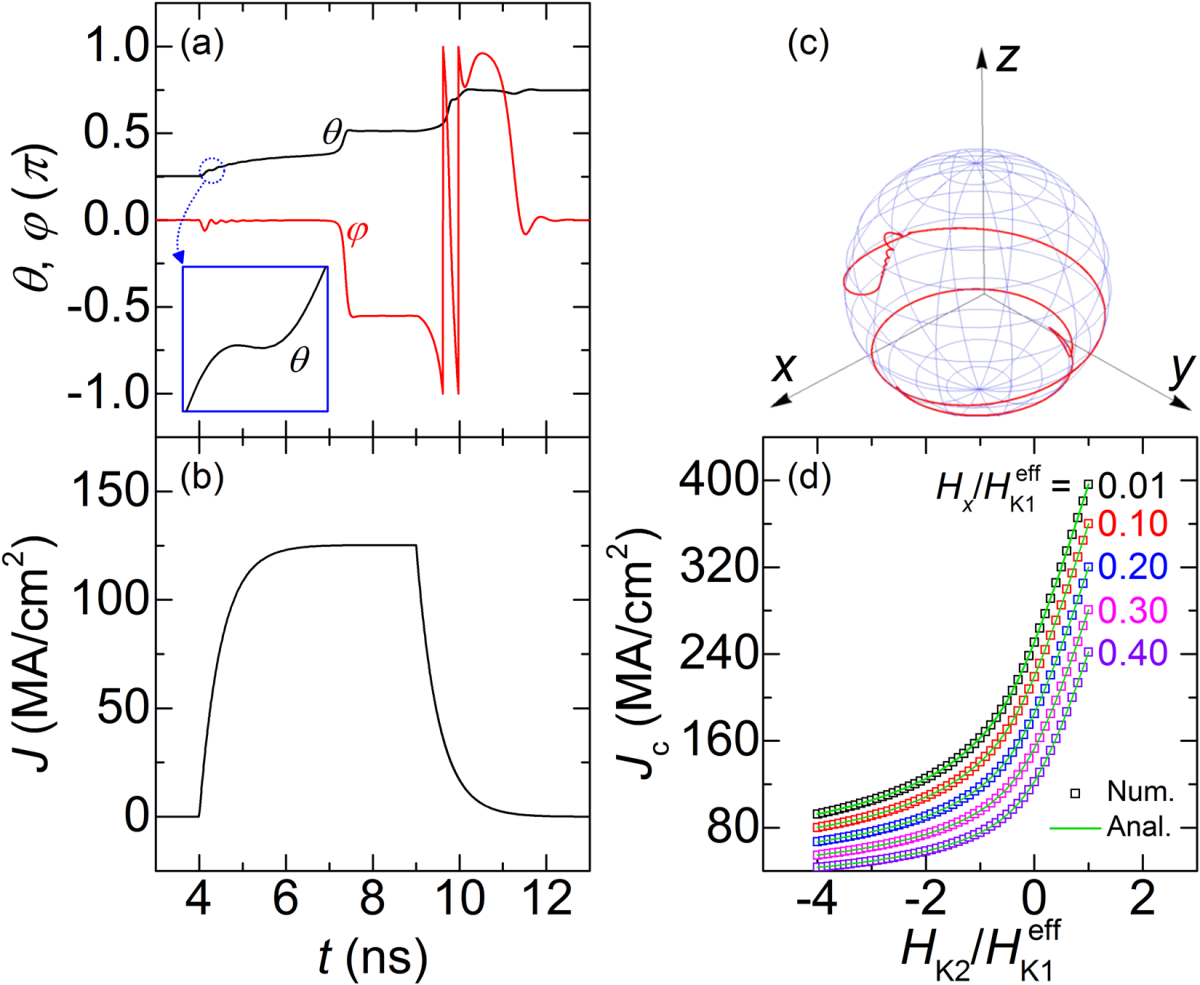
Macrospin simulation results for (**a**) temporal variations of *θ* (black) and *φ* (red) under applied pulse of *J* [in (**b**)] and (**c**) 3D magnetization trajectory. The parameters used are *H*_K1_^eff^ = 5 kOe, *H*_K2_ = −10 kOe, *H*_*x*_ = 0.05 kOe, *M*_S_ = 1000 emu/cm^3^, *α* = 0.1, *γ* = 1.76 × 10^7^ Oe^−1^ s^−1^, *θ*_SH_ = 0.3. The magnified results for the temporal variation of *θ* are shown in the inset of (**a**). (**d**) Results for *J*_c_ as function of *H*_K2_/*H*_K1_^eff^ at various values of *H*_*x*_/*H*_K1_^eff^. Two sets of results are shown: one from the macrospin simulation (symbols) and the other from the analytical expressions derived in this study (lines).

In order to understand the magnetization switching behavior of a system showing the easy-cone state, the macrospin simulation was performed using the following parameters: *H*_K1_^eff^ = 5 kOe, *H*_K2_ = −10 kOe, and *H*_*x*_ = 0.05 kOe. An unusually large value of *H*_K2_ was used for precisely demonstrating its effect on the magnetization switching behavior more clearly. Figure 2(a) shows the macrospin simulation results for the temporal dependences of *θ* and *φ* under an applied pulse *J*, the shape of which is shown in Fig. 2(b). The pulse is on at *t* = 4.0 ns and off at *t* = 9.0 ns, and the variation exhibits an exponential shape with a characteristic time of 0.5 ns. It is observed from Fig. 2(a) that, under a particular pulse cycle, ***m*** is switched from *θ* = ~*θ*_E_ and *φ* = 0 to *θ* = ~(*π* − *θ*_E_) and *φ* = 0. The *φ* value is zero before and after the switching, owing to the application of *H*_*x*_ for the deterministic switching. A detailed switching process is as follows. In the first period of *t* = 4.0–7.0 ns, *θ* increases gradually but *φ* remains nearly unchanged except for some oscillations during the initial stage of the period with their strength decaying with time. In the second period of *t* = 7.0–7.5 ns, an abrupt change in both *θ* and *φ* occurs. The change occurs in the opposite direction, with *θ* in the positive direction and *φ* in the negative direction. Furthermore, the change in *θ* is significantly smaller than that in *φ*. After this abrupt change, both *θ* and *φ* remain nearly unchanged in the third period of *t* = 7.5–9.0 ns; *θ* is slightly higher than *π*/2, but *φ* is slightly lower than −*π*/2. When the pulse is turned off at *t* = 9.0 ns, ***m*** shows a precession motion until it reaches the new equilibrium position. This behavior can be observed more clearly from a 3D illustration of the trajectory of ***m*** *,* as shown in Fig. 2(c).

### Derivations of analytical expressions for critical switching current density

The temporal variations of *θ* and *φ* in the first period of *t* = 4.0–7.0 ns, which are of considerable importance in deriving the analytical expressions for *J*_c_, can be explained by the competition between the precessional torque and damping-like SOT. Provided *φ* ~ 0 during the period, equations (2) and (3) can be rewritten in the form of temporal variations of *θ* and *φ* (refer to Supplementary Section 1 for a detailed derivation):

4 $$\frac{\partial \theta }{\partial t}=-\frac{\gamma \alpha }{1+{\alpha }^{2}}[f(\theta )-{c}_{J}],$$

5 $$\sin \,\theta \frac{\partial \phi }{\partial t}=\frac{\gamma }{1+{\alpha }^{2}}[f(\theta )-{c}_{J}],$$

6 $$f(\theta )\equiv ({H}_{{\rm{K1}}}^{{\rm{eff}}}\,\cos \,\theta +{H}_{{\rm{K2}}}{\cos }^{3}\theta )\sin \,\theta -{H}_{x}\,\cos \,\theta .$$

Here, *f*(*θ*) is the strength of the precessional torque induced by ***H***_eff_. The temporal variation of *θ* can be described by equation (4), whereas that of *φ* can be explained by equation (5), which in a strict sense, is for the *y*-axis component of ∂***m***/∂*t*. It is clear from equations (4) and (5) that the temporal variations of both *θ* and *φ* depend on the difference of the precessional torque (*f*(*θ*)) and the damping-like SOT (*c*_*J*_), with their directions being opposite to each other. In the initial stage of the period where *J* increases rapidly, *c*_*J*_ is dominant over *f*(*θ*), making *f*(*θ*) − *c*_*J*_ < 0. Therefore, *θ* increases, whereas *φ* decreases with time. Considering that *f*(*θ*) increases with increasing *θ*, *f*(*θ*) will start to be dominant over *c*_*J*_, thereby reversing the initial temporal variations. In this stage, *φ* returns to the original position of zero, but *θ* does not; precisely, a closer examination shows that the return path of *θ* is found to be considerably small (refer to the magnified results in the inset of Fig. 2(a)). This is because the *θ* value at which *f*(*θ*) − *c*_*J*_ = 0 continuously increases with the increase of *J*. This process occurs repeatedly until *θ* reaches a critical angle (*θ*_c_) at which *f*(*θ*) is maximum. On reaching *θ*_c_, even a slight increase in *θ* decreases *f*(*θ*) considerably, thereby resulting in the abrupt change in *θ* and *φ* observed in the second period. These temporal variations of *θ* and *φ* may indicate that the SHE-induced switching occurs at *f*(*θ*_c_) − *c*_*J*_ < 0. From this, it is a straightforward task to derive analytical expressions for *J*_c_, which are the central results of this study (refer to Supplementary Section 2 for a detailed derivation):

7 $${J}_{{\rm{c}}}=\frac{2e}{\hslash }\frac{{M}_{{\rm{S}}}{t}_{{\rm{F}}}}{|{\theta }_{{\rm{SH}}}|}f({\theta }_{{\rm{c}}}),$$

8 $$f(\theta )\equiv ({H}_{{\rm{K1}}}^{{\rm{eff}}}\,\cos \,\theta +{H}_{{\rm{K2}}}{\cos }^{3}\theta )\sin \,\theta -|{H}_{x}|\cos \,\theta ,$$

9 $$\begin{array}{cc}\sin {\theta }_{{\rm{c}}} & =\sqrt{\frac{5}{8}-\frac{4+9({H}_{{\rm{K2}}}/{H}_{{\rm{K1}}}^{{\rm{eff}}})}{8[2+\sqrt{4+4({H}_{{\rm{K2}}}/{H}_{{\rm{K1}}}^{{\rm{eff}}})+9{({H}_{{\rm{K2}}}/{H}_{{\rm{K1}}}^{{\rm{eff}}})}^{2}}]}}\\  & \,+\,\frac{(|{H}_{x}|/{H}_{{\rm{K1}}}^{{\rm{eff}}})}{2\sqrt{4+4({H}_{{\rm{K2}}}/{H}_{{\rm{K1}}}^{{\rm{eff}}})+9{({H}_{{\rm{K2}}}/{H}_{{\rm{K1}}}^{{\rm{eff}}})}^{2}}}.\end{array}$$

The analytical expressions for *J*_c_ are rather general in the sense that they can be applied to all structures exhibiting SHE, which comprises NM and FM with PMA. With *H*_K2_ = 0, the *J*_c_ values from the present analytical expressions are identical to those from the equations reported in the literature^27^. The sign of *θ*_SH_ differs depending on the type of NM. The symmetry of the torques, however, is the same whenever the sign of *H*_*x*_ changes together. To consider this feature, absolute values are used for *H*_*x*_ and *θ*_SH_ in equations (7)–(9). Some examples of the NM materials that possess SHE include 3d-, 4d-, and 5d-transition elements (such as Pt, Ta, and W) and alloys (such as CuIr and CuBi)^18,19,23,25,26^. The phase diagram in Fig. 1(b) shows four different types of magnetic states, among which the out-of-plane easy and easy-cone states are of practical importance and occur when the sign of *H*_K1_^eff^ is positive. The window for the former is wider than that for the latter, which occurs only when *H*_K2_/*H*_K1_^eff^ < −1^33,37,38^. It is worth noting that although the phase region for the out-of-plane easy state is predicted by the phenomenological expression of equation (1), its existence is not fully confirmed by the experimental evidence. In order to confirm the validity of the analytical expressions, the results for *J*_c_ obtained from equations (7)–(9) are compared with the numerical results from the macrospin simulation, as shown in Fig. 2(d), where the results for *J*_c_ are shown as a function of *H*_K2_/*H*_K1_^eff^ at various values of *H*_*x*_/*H*_K1_^eff^ ranging from 0.01 to 0.4 (here the *H*_K1_^eff^ value is fixed at 5 kOe). Precisely, in wide ranges of *H*_K2_/*H*_K1_^eff^ and *H*_*x*_/*H*_K1_^eff^, which include both the out-of-plane easy and easy-cone states, the agreement between the two sets of results is excellent, thereby confirming the accuracy of the analytical expressions.

## Discussion

**Figure 3:**
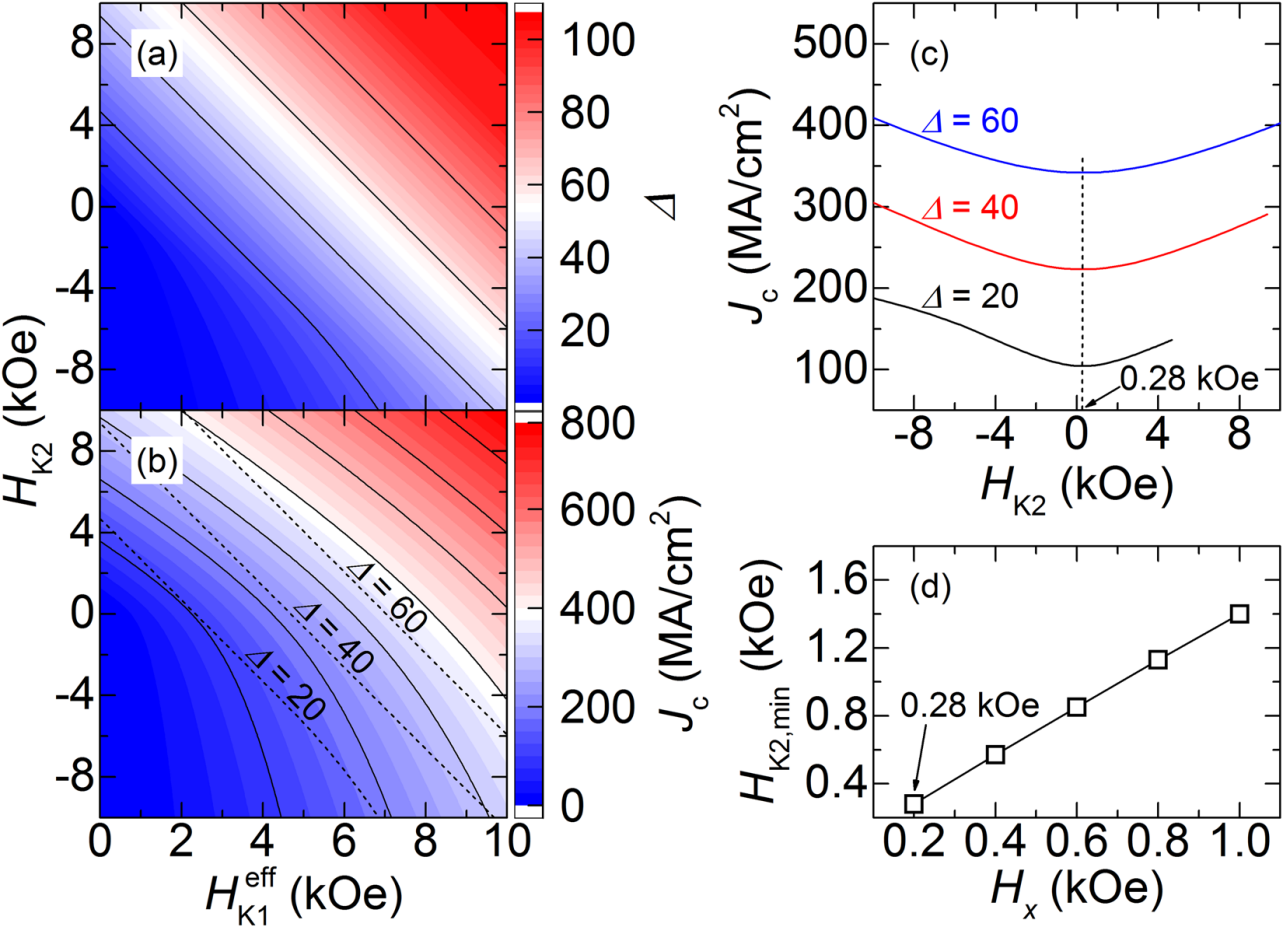
Analytical results for (**a**) Δ and (**b**) *J*_c_ as functions of *H*_K1_^eff^ and *H*_K2_. Contour results for Δ at selected values of 20, 40, and 60 (dotted lines) are also shown in (**b**). (**c**) Analytical results for *J*_c_ as function of *H*_K2_ at three different Δ values of 20, 40, and 60. In all cases, a broad minimum is observed at *H*_K2_ = 0.28 kOe. (**d**) Analytical results for *H*_K2,min_ as function of *H*_*x*_.

The derived analytical expressions can be of considerable value in the design of SHE-based devices, and the results shown in Fig. 3 are one related example. Figures 3 (a) and 3(b) show the contour results for the thermal stability parameter (*Δ*) and *J*_c_ as functions of *H*_K1_^eff^ and *H*_K2_ at a fixed *H*_*x*_ value of 0.2 kOe. The results for *Δ* are calculated using the relation *Δ* = [*E*_PMA_(*π*/2) − *E*_PMA_(*θ*_E_)]/*k*_B_ *T* (*k*_B_ and *T* are the Boltzmann constant and absolute temperature, respectively). It is expected that *Δ* and *J*_c_ scale in a similar manner, and this expectation agrees well with the results in Fig. 3 (a) and 3(b), where both *Δ* and *J*_c_ increase with the increase of *H*_K1_^eff^ and *H*_K2_. A closer examination, however, shows a different tendency for the two parameters. This can be observed clearly in Fig. 3(b) where some of the results for *Δ* (in dotted contours) are superimposed with those for *J*_c_ (solid lines). In fact, the tendency for *Δ* is clearly different from that for *J*_c_ indicating room for design optimization. Along the dotted lines in Fig. 3(b) that show the contour results at fixed values of *Δ* = 20, 40, and 60, the *J*_c_ values change continuously. The results are shown in detail in Fig. 3(c) where the variations of *J*_c_ with *H*_K2_ are shown for the three *Δ* values. For all the *Δ* values, a broad minimum in *J*_c_ is observed at *H*_K2_ ~ 0.28 kOe, which can be an optimized value, at least in terms of *Δ* and *J*_c_, because a small *J*_c_ is preferred at a given *Δ* value. The value of *H*_K2_ showing the minimum *J*_c_ (*H*_K2,min_) can be tuned, as can be shown in Fig. 3(d); in this particular example, *H*_K2,min_ is linearly proportional to *H*_*x*_.

Some examples are demonstrated with the use of the analytical expressions derived in this study to optimize *Δ* and *J*_c_, which are probably the two most important device parameters. The optimized values of *H*_K2_ are small and positive, although they can be tuned by other parameters such as *H*_*x*_. Considering that the sign of *H*_K2_ is negative in typical systems having the out-of-plane easy and easy-cone states, it is rather challenging to develop materials with positive *H*_K2_ values. The origin of *H*_K2_ is still not clear but according to the explanations by Tomas *et al*. and Dieny *et al*., nearly zero or even positive *H*_K2_ values may be accomplished by reducing the interface roughness of NM/FM and FM/tunneling barrier and by forming a proper bulk magnetocrystalline anisotropy in FM^34,35^. In this study, the SHE-induced damping-like torque is only considered, although there are several reports showing that the SOT also can be induced by the Rashba effect^17,20,40–43^. It is, therefore, important to understand the SOT switching induced by the Rashba effect as well as SHE for an FM with *H*_K2_. Investigation toward this direction, which may offer complete understanding on the effects of *H*_K2_ on the SOT switching, is in progress. The pulse duration and rising times can be important parameters affecting the SOT switching. A numerical study performed on a simple PMA system shows that the critical current density for the SOT switching increases with a decrease in the pulse duration time of an in-plane current^28^. A further study on the effects of the pulse rising time on the SOT switching shows that the critical current density is independent of the pulse characteristic time, except that the characteristic time is very short (0.16 ns or lower) (refer to Supplementary Section 3 for detailed results).

In summary, analytical expressions for *J*_c_ induced by SHE were derived in magnetic structures with the first- and second-order perpendicular magnetic anisotropy, and their accuracy was validated by comparing the analytical results with the macrospin simulation results. One example of the usage of the analytical expressions in the design of SOT-MRAM is demonstrated in this study; even at an identical *Δ* value, a minimum in *J*_c_ is observed at slightly positive *H*_K2_ values.

## Methods

For the macrospin simulation, the modified Landau-Liftshitz-Gilbert equation (refer to equations (2) and (3)) including a damping-like SOT was numerically solved using the fourth-order Runge-Kutta method. The following values were used in the simulation: *γ* = 1.76 × 10^7^ O e^−1^ s^−1^; *α* = 0.1; *θ*_SH_ = 0.3; *M*_S_ = 1000 emu/cm^3^. The number of the time step was 5000 and the magnitude for the step-by-step ***m*** displacements was controlled in the range of 0.01 ~ 0.001 by varying the length of the time step.

## Electronic supplementary material

Supplementary materials
