# Supplementary material for: Critical switching current density induced by spin Hall effect in magnetic structures with first- and second-order perpendicular magnetic anisotropy
Source: Sci Rep. 2017 Nov 10;7:15314. doi: 10.1038/s41598-017-15681-2 (PMC5681510; doi:10.1038/s41598-017-15681-2)
Supplement: Supplementary file 1 — Supplementary materials [file 41598_2017_15681_MOESM1_ESM.pdf]

Supplementary Material

Critical switching current density induced by spin Hall effect in magnetic structures with first- and second-order perpendicular magnetic anisotropy

Seok Jin Yun, Kyung-Jin Lee, and Sang Ho Lim*

Department of Materials Science and Engineering, Korea University, Seoul 02841, Korea

October 9, 2017

1. Derivation of equations (4) and (5)

Equation (2) can be reduced by substituting equation (2) into the second term of its right-hand side as follows:

$$\begin{aligned}\frac{\partial \boldsymbol{m}}{\partial t} = & -\frac{\gamma}{1+\alpha^2}\left[\boldsymbol{m}\times\boldsymbol{H}_{\text{eff}} - c_J\,\boldsymbol{m}\times(\boldsymbol{m}\times\hat{y})\right] \\ & -\frac{\gamma\alpha}{1+\alpha^2}\boldsymbol{m}\times\left[\boldsymbol{m}\times\boldsymbol{H}_{\text{eff}} - c_J\,\boldsymbol{m}\times(\boldsymbol{m}\times\hat{y})\right].\end{aligned} \qquad \text{(S1)}$$

Here, $\boldsymbol{m}$ is as follows:

$$\boldsymbol{m} = \sin\theta\cos\varphi\,\hat{x} + \sin\theta\sin\varphi\,\hat{y} + \cos\theta\,\hat{z}. \qquad \text{(S2)}$$

By differentiating equation (S2) with t and substituting $\varphi = 0$, the first derivative of $\boldsymbol{m}$ can be derived as follows:

$$\left.\frac{\partial \boldsymbol{m}}{\partial t}\right|_{\varphi=0} = \sin\theta \frac{\partial \varphi}{\partial t}\hat{y} + \frac{\partial \theta}{\partial t}(\cos\theta\,\hat{x} - \sin\theta\,\hat{z})$$
$$= \sin\theta \frac{\partial \varphi}{\partial t}\hat{y} - \frac{\partial \theta}{\partial t}(\boldsymbol{m}\times\hat{y})|_{\varphi=0} \qquad \text{(S3)}$$

Provided that $\boldsymbol{m} \times \boldsymbol{H}_{\mathrm{eff}}$ and $\boldsymbol{m} \times (\boldsymbol{m} \times \hat{y})$ are $-f(\theta)\hat{y}$ and $-y$ at $\varphi = 0$, respectively, equation (S1) can be manipulated as follows:

$$\left.\frac{\partial \boldsymbol{m}}{\partial t}\right|_{\varphi=0} = \frac{\gamma}{1+\alpha^2}[f(\theta) - c_J]\hat{y} + \frac{\gamma\alpha}{1+\alpha^2}[f(\theta) - c_J](\boldsymbol{m}\times\hat{y})|_{\varphi=0}. \qquad \text{(S4)}$$

Comparing equations (S3) and (S4), equations (4) and (5) are derived.

2. **Derivation of equations (7)−(9)**

As $f(\theta)$ is maximum at θ_c, $\partial f/\partial t$ is zero at this angle.

$$\left.\frac{\partial f}{\partial \theta}\right|_{\theta=\theta_c} = H_{\mathrm{K1}}^{\mathrm{eff}}[1 - 2\sin^2\theta_c + r_{\mathrm{K}}(\cos^4\theta_c - 3\cos^2\theta_c\sin^2\theta_c) + r_x\sin\theta_c] = 0. \qquad \text{(S5)}$$

Here, r_{K} and r_x are $H_{\mathrm{K1}}^{\mathrm{eff}}/H_{\mathrm{K2}}$ and H_x/H_{K2}, respectively. Let us replace X with $\sin\theta$.

$$g(X_c) + r_x X_c = 0, \qquad \text{(S6)}$$
$$g(X) \equiv 1 - 2X^2 + r_{\mathrm{K}}(1 - X^2)^2 - 3r_{\mathrm{K}}(1 - X^2)X^2 \qquad \text{(S7)}$$

By using the quadratic formula, the value of X_{c0} satisfying $g(X) = 0$ can be derived as follows:

$$X_{c0} = \sqrt{\frac{5}{8} - \frac{4 + 9r_K}{8\left(2 + \sqrt{4 + 4r_K + 9{r_K}^2}\right)}}. \tag{S8}$$

Provided that r_x is sufficiently small, the following approximation is applicable.

$$\left.\frac{\partial g}{\partial X}\right|_{X=X_{c0}} \approx \frac{g(X_c) - g(X_{c0})}{X_c - X_{c0}} = \frac{-r_x X_{c0}}{X_c - X_{c0}}. \tag{S9}$$

Please note that $g(X_c) + r_x X_c = 0$ and $g(X_{c0}) = 0$. The left-hand side of equation (S9) can be obtained by substituting equation (S8) into the first derivative of equation (S7).

$$\left.\frac{\partial g}{\partial X}\right|_{X=X_{c0}} = -2X_{c0}\sqrt{4 + 4r_K + 9{r_K}^2}. \tag{S10}$$

From equation (S9), the approximation of X_c is as follows:

$$X_c \approx X_{c0} - r_x X_{c0}\left(\left.\frac{\partial g}{\partial X}\right|_{X=X_{c0}}\right)^{-1}. \tag{S11}$$

By substituting equation (S10) into equation (S11), the approximation of X_c can be derived as follows:

$$\sin\theta_c = X_c \approx X_{c0} + \frac{r_x}{2\sqrt{4 + 4r_K + 9{r_K}^2}}. \tag{S12}$$

Finally, equation (9) is derived by substituting equation (S8) into equation (S12).

3. **Effects of in-plane current pulse characteristic time**

In this study, the in-plane current pulse has an exponential shape with a characteristic time (τ) of 0.5 ns, a typical value achieved from pulse drivers currently in use (refer to Refs. [S1] and [S2]). Left panels of Figs. S1(a)−(c) show the switching state (switching or no switching) as a function of τ and the maximum current density (J_{max}). It is seen from the results that the critical switching current density is independent of the τ, except for $\tau \leq 0.16$ ns where the increase in the SOT is dominant over that in the precessional torque. It is noted that the increase in the precessional torque plays a secondary role in increasing the SOT (refer to the inset of Fig. 2(a)).

4. **Effects of field-like spin-orbit torque**

To consider the field-like torque in the SOT switching simulation, the following equation (S13) is added to equation (2).

$$T_{\mathrm{FL}} = -\gamma\left(\frac{\hbar}{2e}\right)\left(\frac{\zeta_{\mathrm{FL}}J}{M_{\mathrm{S}}t_{\mathrm{F}}}\right)\boldsymbol{m}\times\hat{y}. \tag{S13}$$

where ζ_{FL} is the field-like torque parameter. The right panels of Figs. S1(a)−(c) show the switching state (switching or no switching) as a function of ζ_{FL} and J_{max}. Two features can be noted from the results. First, the critical switching current density tends to decrease with the increase of ζ_{FL}. Second, the switching occurs rather stochastically in the presence of the field-like torque, with this tendency being stronger at higher values of H_{K2}. These results are in agreement with those reported in the literature.[27]

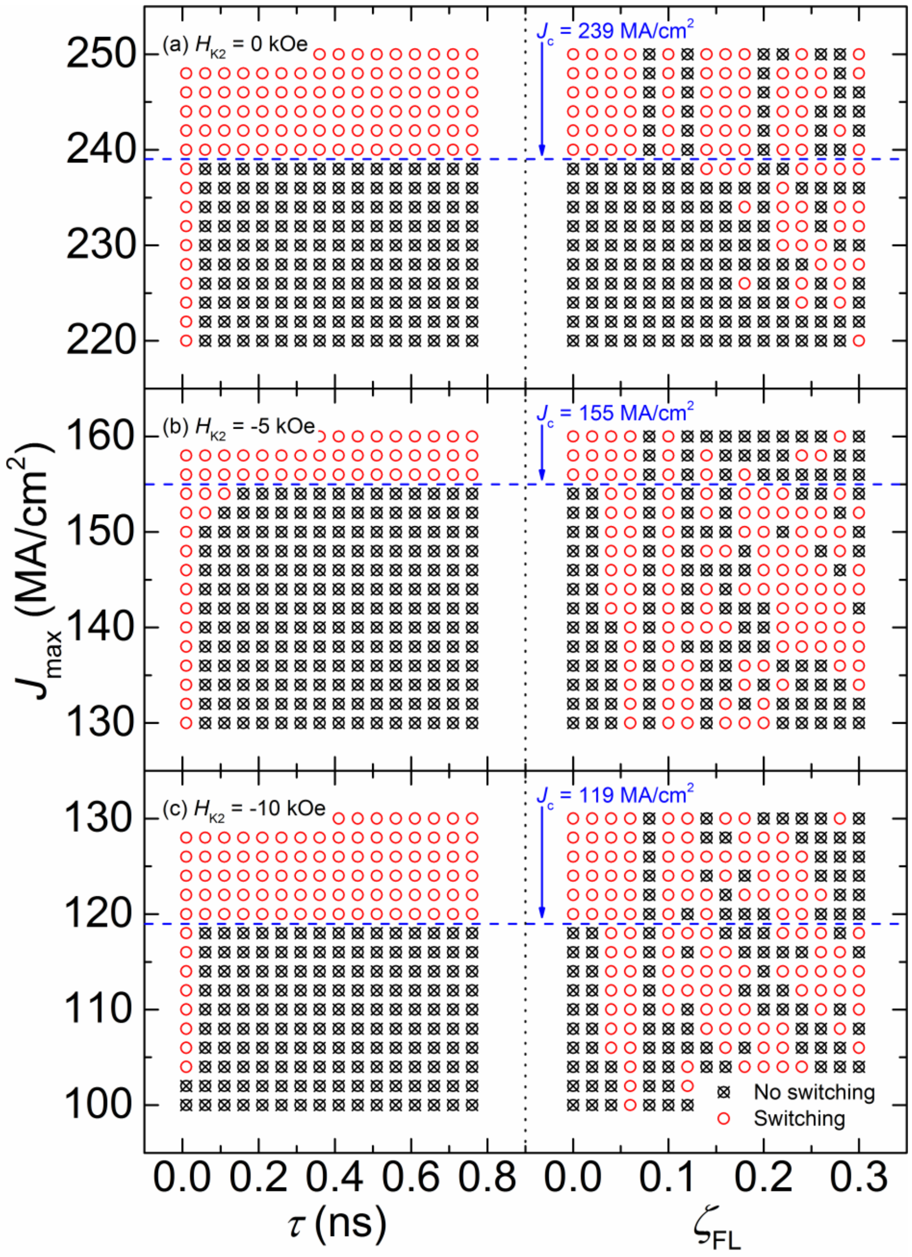

FIG. S1. Macrospin simulation results showing the switching state (switching or no switching) for H_{K2} values of (a) 0 kOe, (b) −5 kOe, and (c) −10 kOe. The left panels show the results as a function of τ and J_{max} and the right panels display the results as a function of ζ_{FL} and J_{max}. The following parameters were used in the simulation: $H_{\mathrm{K1}}{}^{eff}$ = 5 kOe and H_x = 0.2 kOe. The dashed lines indicate the J_c values calculated using the analytical expression.

References

S1. Kim, J.-S., Lee, Y.-K., Lee, J.-S., Shin, Y.-K., Tark, J.-H., Ryu, K.-C. & Yang, B.-D. A fast-switching current-pulse driver for LED backlight. In *Proceedings of IEEE International Symposium on Circuits and Systems*, 1775–1778 (IEEE, Taipei, Taiwan, 2009).

S2. Huang, Y.-C. & Chen, H.-W. A novel fast-switching current-pulse driver for LED backlight applications. In *Proceedings of 2016 International Conference on Consumer Electronics–Taiwan*, 1–2 (IEEE, Nantou, Taiwan, 2016).
